# Evaluation of drug-induced tissue injury by measuring alanine aminotransferase (ALT) activity in silkworm hemolymph

**DOI:** 10.1186/2050-6511-13-13

**Published:** 2012-11-08

**Authors:** Yoshinori Inagaki, Yasuhiko Matsumoto, Keiko Kataoka, Naoya Matsuhashi, Kazuhisa Sekimizu

**Affiliations:** 1 Laboratory of Microbiology, Graduate School of Pharmaceutical Sciences, The University of Tokyo, 7-3-1 Hongo, Bunkyo-ku, Tokyo, 113-0033, Japan; 2 Genome Pharmaceuticals Institute Co., Ltd., The University of Tokyo Entrepreneur Plaza, 7-3-1 Hongo, Bunkyo-ku, Tokyo, 113-0033, Japan

**Keywords:** Silkworm, Alanine aminotransferase, Tissue injury, Animal model

## Abstract

**Background:**

Our previous studies suggest silkworms can be used as model animals instead of mammals in pharmacologic studies to develop novel therapeutic medicines. We examined the usefulness of the silkworm larvae *Bombyx mori* as an animal model for evaluating tissue injury induced by various cytotoxic drugs. Drugs that induce hepatotoxic effects in mammals were injected into the silkworm hemocoel, and alanine aminotransferase (ALT) activity was measured in the hemolymph 1 day later.

**Results:**

Injection of CCl_4_ into the hemocoel led to an increase in ALT activity. The increase in ALT activity was attenuated by pretreatment with *N*-acetyl-_L_-cysteine. Injection of benzoic acid derivatives, ferric sulfate, sodium valproate, tetracycline, amiodarone hydrochloride, methyldopa, ketoconazole, pemoline (Betanamin), *N*-nitroso-fenfluramine, and _D_-galactosamine also increased ALT activity.

**Conclusions:**

These findings indicate that silkworms are useful for evaluating the effects of chemicals that induce tissue injury in mammals.

## Background

Tissue injury induced by chemicals in mammals, including humans, is associated with the rapid development of severe impairment of the organs involved in detoxification, e.g., fulminant hepatic failure [1]. Therefore, assessment of chemical-induced tissue injury is crucial in drug discovery.

In the development of novel therapeutic medicines, *in vivo* trials using animal models are essential for predicting toxicity and drug disposition in the human body. Mice and rats are used to evaluate the toxicity of synthesized compounds and natural medicines [2-4]. The use of mammals for experimental models, however, is associated with a number of problems, such as high cost and ethical issues. An alternative animal model is needed to overcome these problems.

Although invertebrate animals such as *Caenorhabditis elegans* (*C. elegans*) and *Drosophila* larvae have been proposed as model animals for evaluating bacterial pathogenicity and therapeutic effects of antibiotics, their body sizes are too small to inject a fixed amount of sample [5,6]. Large insect larvae can be easily injected into the midgut or subcutaneously with sample solution using a syringe. Silkworm hemolymph and tissue can be harvested separately and used in biochemical, haematological, and immunological analyses [7,8]. Thus, the silkworm is an invertebrate model that can relieve the issues related to the use of mammals and thus promote pharmaceutical studies [7,9-12]. We previously demonstrated that the lethal dose of various cytotoxic substances in silkworms is consistent with that in mammals [7]. Thus, silkworms are considered to be appropriate for evaluating the toxic effects of chemical compounds on animal bodies. In mammals, hepatotoxic substances induce increases in marker enzymes of tissue injury in the blood [13]. Increases in alanine aminotransferase (ALT) activity in mammalian blood are caused by leakage of this enzyme from injured tissue. ALT is conserved throughout evolution [14] and is therefore considered to be a surrogate marker of tissue injury in insect larvae. To date, however, there has been no evidence that ALT activity is increased in the body fluid of the silkworm upon the induction of tissue injury.

The present study aimed to examine ALT activity in the body fluid of silkworm larvae injected with various hepatotoxic compounds. We also analyzed the effectiveness of using the silkworm model for evaluating drugs that have a protective effect against tissue injury induction.

## Methods

### Chemicals

Various cytotoxic drugs were purchased, as follows: carbon tetrachloride (CCl_4_), salicylic acid, ferric sulfate, sodium valproate, *N*-nitroso-fenfluramine, and _D_-galactosamine were purchased from Wako Pure Chemical Industries, Osaka, Japan; acetaminophen was purchased from Tocris Biosciences, Ellisville, MO; acetylsalicylic acid was purchased from Cayman Chemical Co., Ann Arbor, MI; tetracycline was purchased from LKT Laboratories Inc., St Paul, MN; amiodarone hydrochloride was purchased from MP Biomedicals, Solon, OH; methyldopa was purchased from Sawai Pharmaceutical Co., Ltd., Osaka, Japan; ketoconazole was purchased from LKT Laboratories Inc.; and pemoline was purchased from Sanwa Kagaku Kenkyusho Co., Ltd., Nagoya, Japan. *N*-acetyl-_L_-cysteine (NAC), which acts to suppress increases in ALT activity, was purchased from Sigma-Aldrich, St. Louis, MO. Hydrosoluble and liposoluble compounds were dissolved in saline and dimethyl sulfoxide, respectively.

### Animals

Fertilized silkworm eggs (*Bombyx mori*, Hu·Yo × Tukuba·Ne) were purchased from Ehime Sanshu Co., Ltd. (Ehime, Japan). Hatched larvae were fed artificial food, Silkmate 2S (Nosan Corporation, Yokohama, Japan) at 27°C.

### Construction of cytotoxic induction model using silkworm larvae

Fifth-instar silkworm larvae on the first day were fed artificial food, Silkmate 2S, for 1 d. After the body weight increased to 1.8 to 2.2 g, they were fasted for 6 h, and solution containing a cytotoxic compound was injected into the hemocoel from the backside of the larvae. Liposoluble compounds were injected (25 μL/silkworm) using a glass syringe (MICROLITER^TM^ #710, Hamilton Co., Reno, NV) with a 27G needle, and hydrosoluble compounds were injected (50 μL/silkworm) using a disposable syringe (Terumo Corporation, Tokyo, Japan) with a 27G needle. After incubation at 27°C for 1 d, the hemolymph was collected for measurement of ALT activity as described below.

### Examination of suppressive effects against induced cytotoxicity

Fifth-instar silkworm larvae on the first day were fed Silkmate 2S for 1 d. After the body weight increased to 1.8 to 2.2 g, they were fasted for 6 h, and 50 μL of 0.9% saline or 0.4 M NAC was injected into hemocoel from the backside of the larvae using a disposable syringe. After 30 min, 25 μL of olive oil or 15% CCl_4_ was injected into the hemocoel using a glass syringe. After incubation at 27°C for 1 d, the hemolymph was collected for measurement of ALT activity as described below.

### Preparation of tissue homogenates from silkworm larvae

Fifth-instar silkworm larvae on the first day were fed Silkmate 2S for 1 d. After fasting for 6 h, the gut, fat body, silk gland, Malpighian tube, and outer coat were isolated. Each tissue was weighed and homogenized with insect physiologic saline (150 mM NaCl, 5 mM KCl, 1 mM CaCl_2_). Samples were centrifuged at 3000 rpm for 5 min, and the supernatant was collected and stored at −80°C until measurement of ALT activity. The amount of protein in the supernatant was quantified using Lowry’s method.

### Measurement of ALT activity

Five μL of collected hemolymph or the supernatant of homogenized tissue was mixed with 550 μL of a reaction solution containing 0.5 M _L_-alanine, 0.2 mM NADH, 1.3 U/mL lactate dehydrogenase, and 0.9 mg/mL bovine serum albumin. After adding 50 μL of 180 mM 2-oxoglutarate solution, the reaction mixtures were incubated at 30°C for 90 min. Absorbance at 339 nm was recorded to detect decreases in NADH. The slope of the absorbance decrease is proportional to ALT activity. Final ALT activities were determined according to the standard curve drawn from the results of mouse liver homogenate. For ALT activity, 1U was defined as the enzyme activity that forms 1 μmol NAD/min under the assay conditions.

### Statistical analysis

All experiments were performed at least twice and the data are shown as the mean ± standard deviation. The significance of differences was calculated using a 2-tailed Student's *t*-test at the significance level alpha = 0.05.

## Results

### Elevation of ALT activity in the hemolymph of silkworms injected with carbon tetrachloride (CCl_4_)

**Figure 1:**
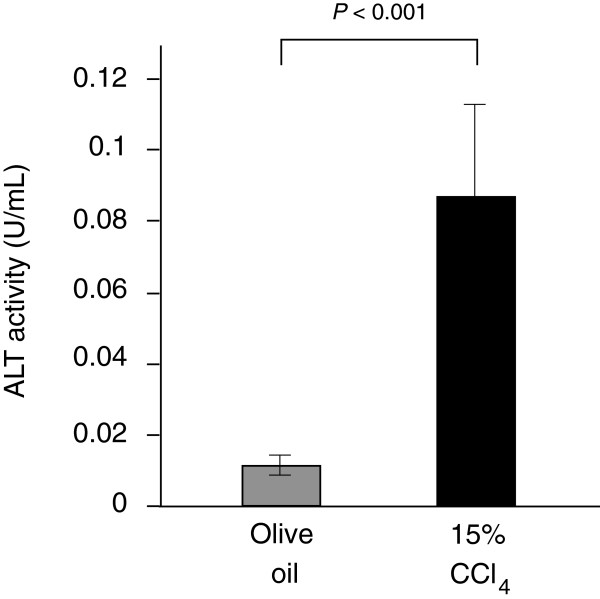
**ALT activity in the silkworm hemolymph injected with CCl**_**4**_**.** Silkworms fasted for 6 h were injected with 15% CCl_4_ or olive oil, and then ALT activity in the silkworm hemolymph was measured 1 d later. (n = 5).

CCl_4_ is generally used as a model compound to evaluate hepatotoxic effects in mammals. Tissue injury induced by CCl_4_ is considered to increase ALT activity in the body fluid. In this study, we examined changes in the ALT activity in silkworm hemolymph after injection of CCl_4_. ALT activity in the silkworm hemolymph increased 8-fold following injection of CCl_4_ compared with injection of olive oil (Figure 1). This finding suggests that tissue injury can be monitored in silkworms by measuring ALT activity. In the subsequent experiments, we used CCl_4_ as a positive control and 0.9% saline or olive oil as a negative control.

### Tissue distribution of ALT activity in silkworms

ALT localizes in the liver and muscles in mammals. Localization of ALT activity in the silkworm has not yet been reported. We determined the tissue distribution of ALT activity to determine which tissue produces ALT activity in the silkworm hemolymph. The total activity and specific activity of ALT in each tissue are shown in Table 1. The highest total activity and the highest specific activity were detected in the gut.

**Table 1:** Tissue distribution of ALT activity in silkworm

**Tissue**	**Total activity**	**Protein**	**Specific activity**
	**(U/tissue)**	**(mg)**	**(U/mg)**
Gut	7.0	4.7	1.5
Fat body	0.2	0.3	0.6
Silk grand	0.4	0.9	0.5
Malpighi grand	0.5	0.2	0.8
Outer coat	3.9	4.9	0.8

### Suppressive effects on ALT activity increases by pretreatment with N-acetyl-L-cysteine (NAC)

**Figure 2:**
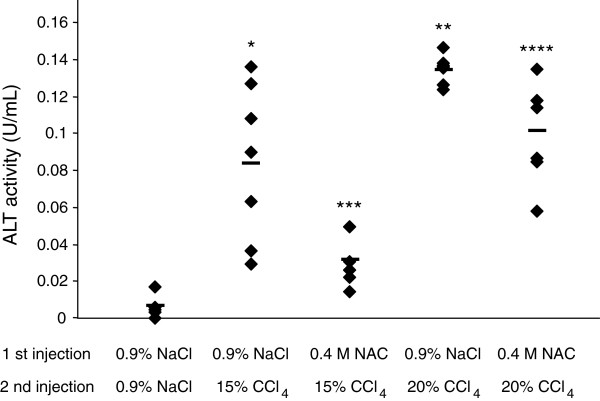
**ALT activity in the silkworm hemolymph injected with 0.4 M NAC prior to CCl**_**4**_**.** Silkworms fasted for 6 h were injected with 0.4 M NAC or 0.9% NaCl (first injection), and then injected with 15% CCl_4_, 20% CCl_4_ or 0.9% NaCl (the second injection) 30 min later. The hemolymph was collected to measure ALT activity after 1 d incubation. The horizontal bar in each line indicates mean ALT activity in each group. *P < 0.01 and **P < 0.001 *vs.* 0.9% NaCl-0.9% NaCl, ***P < 0.01 *vs.* 0.9% NaCl-15% CCl_4_, and ****P < 0.001 *vs.* 0.9% NaCl-20% CCl_4_. (n = 5, 0.9% NaCl-0.9% NaCl; n = 7, other groups).

As described above, CCl_4_ that is hepatotoxic in mammals increased ALT activity in the silkworm hemolymph. Therefore, we considered the silkworm applicable as an animal model to evaluate drug-induced tissue injury. In mammals, radical scavengers such as NAC suppress the cytotoxic effects induced by hepatotoxic substances [15]. Pretreatment with NAC is useful for clarifying whether ALT activity in the silkworm hemolymph is increased due to the production of radicals. In this study, we examined the effect of injecting the silkworm with NAC prior to injection of CCl_4_ on ALT activity in the hemolymph. The ALT activity of CCl_4_-injected silkworms that were preinjected with 0.4 M NAC was much lower than those of silkworms preinjected with 0.9% NaCl (Figure 2).

### Increased ALT activity in the silkworm hemolymph following injection with cytotoxic drugs

**Figure 3:**
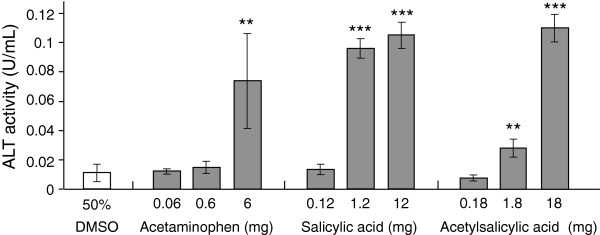
**ALT activity in the silkworm hemolymph injected with benzoic acid derivatives.** Silkworms fasted 6 h were injected with acetaminophen, salicylic acid, or acetylsalicylic acid, and 1 d later the silkworm hemolymph was collected to measure ALT activity. **P < 0.01 and ***P < 0.001 *vs.* negative control. (n = 5).

Consistent with the results described above, CCl_4_ was suggested to induce tissue injury via the production of radicals in the silkworm body. We then examined the influence of benzoic acid derivatives (acetaminophen, salicylic acid, and acetylsalicylic acid), which are known hepatotoxic agents due to the production of radicals by the catalytic reaction of P450 2E1 [16-18], on ALT activity in the silkworm hemolymph. ALT activity in the hemolymph increased in a dose-dependent manner following injection of each of these agents (Figure 3). Compared with the negative control, ALT activity was increased in groups treated with 6 mg acetaminophen (7-fold), 1.2 and 12 mg salicylic acid (9 and 10-fold, respectively), and 1.8 and 18 mg acetylsalicylic acid (3 and 11-fold, respectively). Based on the amount of each drug needed to increase ALT activity, salicylic acid was the most toxic among these three reagents.

**Figure 4:**
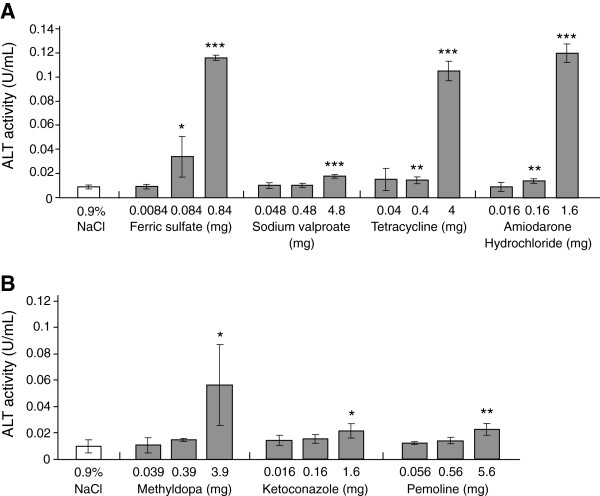
**ALT activity in the silkworm hemolymph injected with various hepatotoxic drugs.** Silkworms fasted 6 h were injected with ferric sulfate, sodium valproate, tetracycline, or amiodarone hydrochloride **(A)**, and with methyldopa, ketoconazole, or pemoline **(B)**, and 1 d later the silkworm hemolymph was collected to measure ALT activity. *P < 0.05, **P < 0.01 and ***P < 0.001 *vs.* negative control. (n = 5).

Substances that produce toxic effects by the production of radicals were suggested to induce tissue injury in the silkworm body and to subsequently increase ALT activity in the silkworm hemolymph. It remains unclear, however, whether tissue injury induced by various biological mechanisms in mammals can be evaluated based on increased ALT activity in the silkworm. Other substances that induce tissue injury in mammals by different mechanisms were also examined to evaluate whether they would induce increases in ALT activity in the silkworm hemolymph. Excessive amounts of iron induce tissue injury by radical production [19]. Sodium valproate, tetracycline, and amiodarone induce tissue damage, probably via inhibiting the function of cell organelles such as mitochondria and lysosomes [20-22]. Compared with the negative control, ALT activity clearly increased in groups treated with 0.084 and 0.84 mg ferric acid (4 and 13-fold, respectively), 4.8 mg sodium valproate (2-fold), 0.4 and 4 mg tetracycline (2 and 12-fold, respectively), and 0.16 and 1.6 mg amiodarone hydrochloride (2 and 13-fold, respectively; Figure 4A).

We then examined the induction of tissue injury by methyldopa, ketoconazole, and pemoline (Betanamin) in the silkworm. These agents are thought to induce hepatic injury in mammals by the formation of metabolites that cause immune hypersensitivity, such as eosinophilia [23-25]. All of the reagents increased ALT activity (3.9 mg methyldopa, 6-fold; 1.6 mg ketoconazole, 2-fold; and 5.6 mg pemoline, 2-fold; Figure 4B).

**Figure 5:**
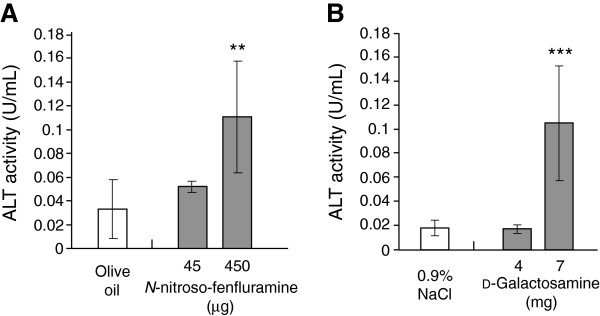
**ALT activity in the silkworm hemolymph injected with*N*-nitroso-fenfluramine and**_**D**_**-galactosamine.** Silkworms fasted 6 h were injected with *N*-nitroso-fenfluramine **(A)**, and with injected _D_-galactosamine **(B)**, and 1 d later the silkworm hemolymph was collected to measure ALT activity. **P < 0.01 and ***P < 0.001 *vs.* negative control. (n = 5).

Excessive intake of *N*-nitroso-fenfluramine, which is generally used as a food supplement, has hepatotoxic effects [26,27]. Rapid depletion of ATP by impaired mitochondrial function and induction of DNA damage by the production of alkyl cations are thought to be the mechanism of *N*-nitroso-fenfluramine-induced cell death [28]. We tested whether silkworms can be used to detect *N*-nitroso-fenfluramine-induced tissue injury by measuring ALT activity in the hemolymph. ALT activity in the hemolymph of silkworms injected with 450 μg *N*-nitroso-fenfluramine increased 3-fold (Figure 5A).

_D_-Galactosamine is a hepatotoxin that induces the depletion of uridine with subsequent necrosis [29]. This compound is frequently used for the construction of fulminant hepatic injury models [29]. Inhibition of the synthesis of nucleic acids, proteins, and lipids by UDP-glucosamine, which is derived from _D_-galactosamine, is the suggested mechanism of tissue damage [29]. We examined whether tissue injury was induced in silkworms by injection of _D_-galactosamine. ALT activity in silkworm larvae injected with 7 mg _D_-galactosamine was increased compared with the negative control (injected 0.9% saline, 6-fold difference; Figure 5B).

## Discussion

The findings of the present study demonstrate the applicability of silkworm larvae as an animal model for evaluating drug-induced tissue injury based on measurements of ALT activity in the hemolymph. ALT activity levels in human blood are considered to be a highly sensitive and fairly specific preclinical and clinical biomarker of cytotoxicity or hepatotoxicity [13]; therefore, ALT activity levels in the blood of mammals are measured in many pharmaceutical studies to evaluate the hepatotoxic effects induced by natural products or newly synthesized chemicals. Here, we demonstrated that ALT activity levels were increased in silkworm larvae by the injection of various cytotoxic drugs into the hemocoel. The results strongly suggest that we could establish a new experimental model to evaluate tissue injury effects using silkworm larvae.

The silkworm has been progressively developed as a scientifically useful experimental animal model [30]. Established silkworm models of infection with pathogenic bacteria and true fungi have been used to evaluate the effects of antibiotics and identify novel virulence genes [31-35]. The established hyperglycemic silkworm model is effective for developing antidiabetic drugs [36]. These studies suggest that silkworms can be used as model animals instead of mammals, such as mice and rats, in pharmacologic studies to develop novel therapeutic medicines. Furthermore, silkworms and mammals have common metabolic pathways involving cytochrome P450s and conjugation enzymes [7]. Cytotoxic effects on tissue and subsequent processes such as the release of marker enzymes from damaged cells occur similarly in silkworms and mammals. In the present study, we showed that ALT activity levels in the silkworm hemolymph were increased by the administration of CCl_4_. In addition, the increase in ALT activity induced by CCl_4_ administration was suppressed by pretreatment with NAC, suggesting that NAC suppressed CCl_4_-induced tissue injury. NAC is a radical scavenger that attenuates hepatotoxic effects induced in the mammalian liver and is used to treat patients with acute acetaminophen hepatotoxicity [15,37]. The present result revealed that NAC has similar suppressive effects in the silkworm body. This silkworm model is thus considered to be useful not only for analyzing the histotoxicity of compounds, but also for the discovery of drugs that have protective effects against histotoxicity. Although we demonstrated the tissue distribution of ALT activity in the silkworm, the mechanism of tissue injury induction detected by elevated ALT levels remains unclear. The present silkworm model can be used to rapidly evaluate histotoxicity, but is not sufficient to elucidate the specific target of drugs. Further studies are needed to clarify the mechanism of tissue injury induction in silkworm.

The prediction of drug hepatotoxicity is crucial for drug discovery and development. Although small mammals such as mice and rats are generally used to evaluate hepatotoxicity, their use is associated with several problems, such as high experimental costs and ethical issues. *In vitro* assay systems using human hepatocytes have been developed in an attempt to solve these problems [38-40]. Toxicogenomic systems are suggested to be effective for predicting hepatotoxicity according to the varied expression of hepatotoxicity-responsive genes [41-43]. The collection of mammalian cells as a material and the conditional differences from *in vivo* examination, however, remain problems in these *in vitro* assay systems. The silkworm tissue injury model established in the present study is a new animal model of histotoxicity. According to the tissue distribution of ALT activity, the gut had the highest ALT activity among other tissues in the silkworm. Thus, in the silkworm, increased ALT activity appears to be induced by tissue injury in the gut. This silkworm model would be extremely useful for evaluating the histotoxicity of newly synthesized chemicals prior to using mice or rats. We expect that the number of mammals needed for drug development can be reduced by first using the silkworm model.

## Conclusions

The present study showed that ALT activity in the silkworm hemolymph is increased by the injection of various cytotoxic drugs. The present silkworm model is applicable for evaluating the toxicity of newly synthesized compounds. This method is more sensitive than toxicity assays based on counting the number of surviving silkworms after administration of test samples. Although further validation and applied research using other types of compounds must be performed, the use of this silkworm model prior to the use of mammals partially addresses the ethical and financial issues related to animal experiments using mammals.

## Abbreviations

ALT: Alanine aminotransferase; *C. elegans*: *Caenorhabditis elegans*.

## Competing interests

The authors and Genome Pharmaceuticals Institute Co., Ltd (Tokyo, Japan) declare that they have no competing interests. Employment costs for silkworm rearing were partially supported by Genome Pharmaceuticals Institute Co., Ltd (Tokyo, Japan).

## Authors’ contributions

KK and NM performed silkworm toxic assay. YI, YM and KS conceived the study and coordinated the writing of the manuscript. All authors read and approved the final manuscript.

## Pre-publication history

The pre-publication history for this paper can be accessed here:

http://www.biomedcentral.com/2050-6511/13/13/prepub
